# Supplementary material for: Differential metabolic activity and discovery of therapeutic targets using summarized metabolic pathway models
Source: NPJ Syst Biol Appl. 2019 Mar 1;5:7. doi: 10.1038/s41540-019-0087-2 (PMC6397295; doi:10.1038/s41540-019-0087-2)
Supplement: Supplementary file 1 — Supplementary Tables and figures. [file 41540_2019_87_MOESM1_ESM.pdf]

Module	Main Category	Sub category of main category (if exist)	Description/Name	KGML	Paths	module_link	start_molecule	end_molecule
M00001	Carbohydrate and lipid metabolism	Central carbohydrate metabolism	Glycolysis (Embden-Meyerhof pathway), glucose => pyruvate	hsa00010	hsa01200;hsa00010	http://www.genome	C00267: alpha-D-Glucose	C00022: Pyruvate
M00002	Carbohydrate and lipid metabolism	Central carbohydrate metabolism	Glycolysis, core module involving three-carbon compounds	hsa00010	hsa01200;hsa01230;hsa00010	http://www.genome	C00111: Glycerone phosphate	C00022: Pyruvate
M00003	Carbohydrate and lipid metabolism	Central carbohydrate metabolism	Gluconeogenesis, oxaloacetate => fructose-6P	hsa00010	hsa00010;hsa00020	http://www.genome	C00036: Oxaloacetate	C05345: beta-D-Fructose 6-phosphate
M00004	Carbohydrate and lipid metabolism	Central carbohydrate metabolism	Pentose phosphate pathway (Pentose phosphate cycle)	hsa00030	hsa01200;hsa00030	http://www.genome	C01172: beta-D-Glucose 6-phosphate	C01172: beta-D-Glucose 6-phosphate
M00006	Carbohydrate and lipid metabolism	Central carbohydrate metabolism	Pentose phosphate pathway, oxidative phase, glucose 6P => ribulose 5P	hsa00030	hsa01200;hsa00030	http://www.genome	C01172: beta-D-Glucose 6-phosphate	C00199: D-Ribulose 5-phosphate
M00007	Carbohydrate and lipid metabolism	Central carbohydrate metabolism	Pentose phosphate pathway, non-oxidative phase, fructose 6P => ribose	hsa00030	hsa01200;hsa01230;hsa00030	http://www.genome	C05345+C00118: beta-D-Fructose 6-phosphate + D-Glyceraldehyde 3-phos	C00117: D-Ribose 5-phosphate
M00009	Carbohydrate and lipid metabolism	Central carbohydrate metabolism	Citrate cycle (TCA cycle, Krebs cycle)	hsa00020	hsa01200;hsa00020	http://www.genome	C00024+C00036: Acetyl-CoA + Oxaloacetate	C00036: Oxaloacetate
M00010	Carbohydrate and lipid metabolism	Central carbohydrate metabolism	Citrate cycle, first carbon oxidation, oxaloacetate => 2-oxoglutarate	hsa00020	hsa01200;hsa01210;hsa01230;hsa00020	http://www.genome	C00036+C00024: Oxaloacetate + Acetyl-CoA	C00026: 2-Oxoglutarate
M00011	Carbohydrate and lipid metabolism	Central carbohydrate metabolism	Citrate cycle, second carbon oxidation, 2-oxoglutarate => oxaloacetate	hsa00020	hsa01200;hsa00020	http://www.genome	C00026+C15972: 2-Oxoglutarate + Enzyme N6-(lipoyl)lysine	C00036: Oxaloacetate
M00013	Carbohydrate and lipid metabolism	Other carbohydrate metabolism	Malonate semialdehyde pathway, propanoyl-CoA => acetyl-CoA	hsa00640	hsa01200;hsa00640	http://www.genome	C00100: Propanoyl-CoA	C00024: Acetyl-CoA
M00014	Carbohydrate and lipid metabolism	Other carbohydrate metabolism	Glucuronate pathway (uronate pathway)	hsa00040	hsa00040	http://www.genome	C00029: UDP-glucose	C00231: D-Xylulose 5-phosphate
M00015	Nucleotide and amino acid metabolism	Arginine and proline metabolism	Proline biosynthesis, glutamate => proline	hsa00330	hsa01230;hsa00330	http://www.genome	C00025: L-Glutamate	C00148: L-Proline
M00020	Nucleotide and amino acid metabolism	Serine and threonine metabolism	Serine biosynthesis, glycerate-3P => serine	hsa00260	hsa01200;hsa01230;hsa00260	http://www.genome	C00197: 3-Phospho-D-glycerate	C00065: L-Serine
M00027	Nucleotide and amino acid metabolism	Other amino acid metabolism	GABA (gamma-Aminobutyrate) shunt	hsa00250	hsa00250	http://www.genome	C00025: L-Glutamate	C00042: Succinate
M00029_1	Nucleotide and amino acid metabolism	Arginine and proline metabolism	Urea cycle	hsa00220	hsa01230;hsa00220	http://www.genome	C00014+C00049: Ammonia + L-Aspartate	C00122: Fumarate
M00029_2	Nucleotide and amino acid metabolism	Arginine and proline metabolism	Urea cycle	hsa00220	hsa01230;hsa00220	http://www.genome	C00014+C00049: Ammonia + L-Aspartate	C00086: Urea
M00032	Nucleotide and amino acid metabolism	Lysine metabolism	Lysine degradation, lysine => saccharopine => acetoacetyl-CoA	hsa00310	hsa00310	http://www.genome	C00047: L-Lysine	C00332: Acetoacetyl-CoA
M00034_1	Nucleotide and amino acid metabolism	Cysteine and methionine metabolism	Methionine salvage pathway	hsa00270	hsa00270	http://www.genome	C00073: L-Methionine	C00147: Adenine
M00035_1	Nucleotide and amino acid metabolism	Cysteine and methionine metabolism	Methionine degradation	hsa00270	hsa00270	http://www.genome	C00073+C00065: L-Methionine + L-Serine	C02291: L-Cystathionine
M00036	Nucleotide and amino acid metabolism	Branched-chain amino acid metabolism	Leucine degradation, leucine => acetoacetate + acetyl-CoA	hsa00280	hsa00280	http://www.genome	C00123: L-Leucine	C00164: Acetoacetate
M00037	Nucleotide and amino acid metabolism	Aromatic amino acid metabolism	Melatonin biosynthesis, tryptophan => serotonin => melatonin	hsa00380	hsa00380	http://www.genome	C00078: L-Tryptophan	C01598: Melatonin
M00042	Nucleotide and amino acid metabolism	Aromatic amino acid metabolism	Catecholamine biosynthesis, tyrosine => dopamine => noradrenaline =>	hsa00350	hsa00350	http://www.genome	C00082: L-Tyrosine	C00788: L-Adrenaline
M00043	Nucleotide and amino acid metabolism	Aromatic amino acid metabolism	Thyroid hormone biosynthesis, tyrosine => triiodothyronine/thyroxine	hsa00350	hsa00350	http://www.genome	C00082: L-Tyrosine	C02465: Triiodothyronine
M00044_1	Nucleotide and amino acid metabolism	Aromatic amino acid metabolism	Tyrosine degradation, tyrosine => homogentisate	hsa00350	hsa00350	http://www.genome	C00082: L-Tyrosine	C00122: Fumarate
M00046_1	Nucleotide and amino acid metabolism	Pyrimidine metabolism	Pyrimidine degradation, uracil => beta-alanine, thymine => 3-aminoisobu	hsa00240	hsa00240	http://www.genome	C00106: Uracil	C00099: beta-Alanine
M00046_2	Nucleotide and amino acid metabolism	Pyrimidine metabolism	Pyrimidine degradation, uracil => beta-alanine, thymine => 3-aminoisobu	hsa00240	hsa00240	http://www.genome	C00178: Thymine	C05145: 3-Aminoisobutyric acid
M00047	Nucleotide and amino acid metabolism	Other amino acid metabolism	Creatine pathway	hsa00330	hsa00330	http://www.genome	C00062: L-Arginine	C00791: Creatinine
M00048	Nucleotide and amino acid metabolism	Purine metabolism	Inosine monophosphate biosynthesis, PRPP + glutamine => IM	hsa00230	hsa00230	http://www.genome	C00119+C00064: 5-Phospho-alpha-D-ribose 1-diphosphate + L-Glutamine	C00130: IMP
M00049	Nucleotide and amino acid metabolism	Purine metabolism	Adenine ribonucleotide biosynthesis, IMP => ADP,ATP	hsa00230	hsa00230	http://www.genome	C00130: IMP	C00002: ATP
M00050	Nucleotide and amino acid metabolism	Purine metabolism	Guanine ribonucleotide biosynthesis IMP => GDP,GTP	hsa00230	hsa00230	http://www.genome	C00130: IMP	C00044: GTP
M00051_1	Nucleotide and amino acid metabolism	Pyrimidine metabolism	Uridine monophosphate biosynthesis, glutamine (+ PRPP) => UMP	hsa00240	hsa00240	http://www.genome	C00064+C00119: L-Glutamine + 5-Phospho-alpha-D-ribose 1-diphosphate	C00105: UMP
M00052	Nucleotide and amino acid metabolism	Pyrimidine metabolism	Pyrimidine ribonucleotide biosynthesis, UMP => UDP/UTP,CDP/CTP	hsa00240	hsa00240	http://www.genome	C00105: UMP	C00112: CDP
M00055	Carbohydrate and lipid metabolism	Glycan metabolism	N-glycan precursor biosynthesis	hsa00510	hsa00510	http://www.genome	C00110: Dolichyl phosphate	G00008:
M00056_1	Carbohydrate and lipid metabolism	Glycan metabolism	O-glycan biosynthesis, mucin type core	hsa00512	hsa00512	http://www.genome	C02189+G10611: [Protein]-L-serine	G00025:
M00056_2	Carbohydrate and lipid metabolism	Glycan metabolism	O-glycan biosynthesis, mucin type core	hsa00512	hsa00512	http://www.genome	C02189+G10611: [Protein]-L-serine	G00029:
M00056_3	Carbohydrate and lipid metabolism	Glycan metabolism	O-glycan biosynthesis, mucin type core	hsa00512	hsa00512	http://www.genome	C02189+G10611: [Protein]-L-serine	G00031:
M00057	Carbohydrate and lipid metabolism	Glycosaminoglycan metabolism	Glycosaminoglycan biosynthesis, linkage tetrasaccharide	hsa00532	hsa00532	http://www.genome	C02189: [Protein]-L-serine	G00157:
M00058	Carbohydrate and lipid metabolism	Glycosaminoglycan metabolism	Glycosaminoglycan biosynthesis, chondroitin sulfate backbone	hsa00532	hsa00532	http://www.genome	G00157:	G00160:
M00059	Carbohydrate and lipid metabolism	Glycosaminoglycan metabolism	Glycosaminoglycan biosynthesis, heparan sulfate backbone	hsa00534	hsa00534	http://www.genome	G00157:	G00164:
M00065	Carbohydrate and lipid metabolism	Glycan metabolism	GPI-anchor biosynthesis, core oligosaccharide	hsa00563	hsa00563	http://www.genome	C01194: 1-Phosphatidyl-D-myo-inositol	G13044:
M00066	Carbohydrate and lipid metabolism	Lipid metabolism	Lactosylceramide biosynthesis	hsa00600	hsa00600	http://www.genome	C00195: N-Acylsphingosine	C01290: beta-D-Galactosyl-(1->4)-beta-D-glucosyl-(11)-c
M00067_1	Carbohydrate and lipid metabolism	Lipid metabolism	Cerebroside and sulfatide biosynthesis	hsa00600	hsa00600;hsa00565	http://www.genome	C00195: N-Acylsphingosine	C06125: Sulfatide
M00067_2	Carbohydrate and lipid metabolism	Lipid metabolism	Cerebroside and sulfatide biosynthesis	hsa00600	hsa00600;hsa00565	http://www.genome	C03201: 1-Alkyl-2-acylglycerol	C20825: Seminolipid
M00068	Carbohydrate and lipid metabolism	Glycan metabolism	Glycosphingolipid biosynthesis, globo-series, LacCer => Gb4Cer	hsa00603	hsa00603	http://www.genome	G00092:	G00094:
M00069	Carbohydrate and lipid metabolism	Glycan metabolism	Glycosphingolipid biosynthesis, ganglio series, LacCer => GT3	hsa00604	hsa00604	http://www.genome	G00092:	G00118:
M00070	Carbohydrate and lipid metabolism	Glycan metabolism	Glycosphingolipid biosynthesis, lacto-series, LacCer => Lc4Cer	hsa00601	hsa00601	http://www.genome	G00092:	G00037:
M00071	Carbohydrate and lipid metabolism	Glycan metabolism	Glycosphingolipid biosynthesis, neolacto-series, LacCer => nLc4Cer	hsa00601	hsa00601	http://www.genome	G00092:	G00050:
M00073	Carbohydrate and lipid metabolism	Glycan metabolism	N-glycan precursor trimming	hsa00510	hsa00510	http://www.genome	G00009:	G00012:
M00075_1	Carbohydrate and lipid metabolism	Glycan metabolism	N-glycan biosynthesis, complex type	hsa00510	hsa00510	http://www.genome	G00013:	G00019:
M00075_2	Carbohydrate and lipid metabolism	Glycan metabolism	N-glycan biosynthesis, complex type	hsa00510	hsa00510	http://www.genome	G00013:	G00022:
M00075_3	Carbohydrate and lipid metabolism	Glycan metabolism	N-glycan biosynthesis, complex type	hsa00510	hsa00510	http://www.genome	G00013:	G00018:
M00076	Carbohydrate and lipid metabolism	Glycosaminoglycan metabolism	Dermatan sulfate degradation	hsa00531	hsa00531	http://www.genome	C00426: Dermatan sulfate	G00872:
M00077	Carbohydrate and lipid metabolism	Glycosaminoglycan metabolism	Chondroitin sulfate degradation	hsa00531	hsa00531	http://www.genome	G12336:	G00872:
M00078	Carbohydrate and lipid metabolism	Glycosaminoglycan metabolism	Heparan sulfate degradation	hsa00531	hsa00531	http://www.genome	C00925: Heparan sulfate	G02632:
M00079	Carbohydrate and lipid metabolism	Glycosaminoglycan metabolism	Keratan sulfate degradation	hsa00531	hsa00531	http://www.genome	C00573: Keratan sulfate	G01391:
M00082	Carbohydrate and lipid metabolism	Fatty acid metabolism	Fatty acid biosynthesis, initiation	hsa00061	hsa01212;hsa00061	http://www.genome	C00024: Acetyl-CoA	C05744: Acetoacetyl-[acp]
M00083	Carbohydrate and lipid metabolism	Fatty acid metabolism	Fatty acid biosynthesis, elongation	hsa00061	hsa01212;hsa00061	http://www.genome	C03939: Acetyl-[acyl-carrier protein]	C05745: Butyryl-[acp]
M00085	Carbohydrate and lipid metabolism	Fatty acid metabolism	Fatty acid biosynthesis, elongation, mitochondria	hsa00062	hsa01212;hsa00062	http://www.genome	C00024: Acetyl-CoA	C00040: Acyl-CoA
M00087	Carbohydrate and lipid metabolism	Fatty acid metabolism	beta-Oxidation	hsa00071	hsa01212;hsa00071	http://www.genome	C00154: Palmitoyl-CoA	C02593: Tetradecanoyl-CoA
M00089	Carbohydrate and lipid metabolism	Fatty acid metabolism	Triacylglycerol biosynthesis	hsa00561	hsa00561	http://www.genome	C00093: sn-Glycerol 3-phosphate	C00422: Triacylglycerol
M00090	Carbohydrate and lipid metabolism	Fatty acid metabolism	Phosphatidylcholine (PC) biosynthesis, choline => PC	hsa00564	hsa00564	http://www.genome	C00114: Choline	C00157: Phosphatidylcholine
M00091	Carbohydrate and lipid metabolism	Fatty acid metabolism	Phosphatidylcholine (PC) biosynthesis, PE => PC	hsa00564	hsa00564	http://www.genome	C00350: Phosphatidylethanolamine	C00157: Phosphatidylcholine
M00092	Carbohydrate and lipid metabolism	Fatty acid metabolism	Phosphatidylethanolamine (PE) biosynthesis, ethanolamine => PE	hsa00564	hsa00564	http://www.genome	C00189: Ethanolamine	C00350: Phosphatidylethanolamine
M00094_1	Carbohydrate and lipid metabolism	Lipid metabolism	Ceramide biosynthesis	hsa00600	hsa00600	http://www.genome	C00154+C00065: Palmitoyl-CoA + L-Serine	C00195: N-Acylsphingosine
M00095	Carbohydrate and lipid metabolism	Terpenoid backbone biosynthesis	C5 isoprenoid biosynthesis, mevalonate pathway	hsa00900	hsa00900	http://www.genome	C00024: Acetyl-CoA	C00235: Dimethylallyl diphosphate
M00098	Carbohydrate and lipid metabolism	Lipid metabolism	Acylglycerol degradation	hsa00561	hsa00561	http://www.genome	C00422: Triacylglycerol	C00116: Glycerol
M00099	Carbohydrate and lipid metabolism	Lipid metabolism	Sphingosine biosynthesis	hsa00600	hsa00600	http://www.genome	C00154: Palmitoyl-CoA	C00319: Sphingosine
M00100	Carbohydrate and lipid metabolism	Lipid metabolism	Sphingosine degradation	hsa00600	hsa00600	http://www.genome	C00319: Sphingosine	C00346: Ethanolamine phosphate
M00101	Carbohydrate and lipid metabolism	Sterol biosynthesis	Cholesterol biosynthesis, squalene 2,3-epoxide => cholesterol	hsa00100	hsa00100	http://www.genome	C01054: (S)-2,3-Epoxysqualene	C00187: Cholesterol
M00103	Carbohydrate and lipid metabolism	Sterol biosynthesis	Cholecalciferol biosynthesis	hsa00100	hsa00100	http://www.genome	C01164: 7-Dehydrocholesterol	C01673: Calcitriol
M00104_1	Carbohydrate and lipid metabolism	Sterol biosynthesis	Bile acid biosynthesis, cholesterol => cholate	hsa00120	hsa00120	http://www.genome	C00187: Cholesterol	C00695: Cholic acid
M00104_2	Carbohydrate and lipid metabolism	Sterol biosynthesis	Bile acid biosynthesis, cholesterol => cholate	hsa00120	hsa00120	http://www.genome	C00187: Cholesterol	C02528: Chenodeoxycholate
M00106_1	Carbohydrate and lipid metabolism	Sterol biosynthesis	Conjugated bile acid biosynthesis, cholate => taurocholate/glycocholate	hsa00120	hsa00120	http://www.genome	C00695: Cholic acid	C05122: Taurocholate
M00106_2	Carbohydrate and lipid metabolism	Sterol biosynthesis	Conjugated bile acid biosynthesis, cholate => taurocholate/glycocholate	hsa00120	hsa00120	http://www.genome	C00695: Cholic acid	C01921: Glycocholate
M00107	Carbohydrate and lipid metabolism	Sterol biosynthesis	Steroid hormone biosynthesis, cholesterol => prognenolone => progester	hsa00140	hsa00140	http://www.genome	C00187: Cholesterol	C00410: Progesterone
M00108	Carbohydrate and lipid metabolism	Sterol biosynthesis	C21-Steroid hormone biosynthesis, progesterone => corticosterone/aldos	hsa00140	hsa00140	http://www.genome	C00410: Progesterone	C01780: Aldosterone
M00109	Carbohydrate and lipid metabolism	Sterol biosynthesis	C21-Steroid hormone biosynthesis, progesterone => cortisol/cortisone	hsa00140	hsa00140	http://www.genome	C00410: Progesterone	C00762: Cortisone
M00110	Carbohydrate and lipid metabolism	Sterol biosynthesis	C19/C18-Steroid hormone biosynthesis, pregnenolone => androstenedio	hsa00140	hsa00140	http://www.genome	C01953: Pregnenolone	C00468: Estrone
M00118	Nucleotide and amino acid metabolism	Cofactor and vitamin biosynthesis	Glutathione biosynthesis, glutamate => glutathione	hsa00480	hsa00480	http://www.genome	C00025: L-Glutamate	C00051: Glutathione
M00120	Nucleotide and amino acid metabolism	Cofactor and vitamin biosynthesis	Coenzyme A biosynthesis, pantothenate => CoA	hsa00770	hsa00770	http://www.genome	C00864: Pantothenate	C00010: CoA
M00128	Nucleotide and amino acid metabolism	Cofactor and vitamin biosynthesis	Ubiquinone biosynthesis, eukaryotes, 4-hydroxybenzoate => ubiquinone	hsa00130	hsa00130	http://www.genome	C00156: 4-Hydroxybenzoate	C00399: Ubiquinone
M00130	Carbohydrate and lipid metabolism	Lipid metabolism	Inositol phosphate metabolism, PI=> PIP2 => Ins(1,4,5)P3 => Ins(1,3,4,!	hsa00562	hsa00562	http://www.genome	C01194: 1-Phosphatidyl-D-myo-inositol	C01272: 1D-myo-Inositol 1,3,4,5-tetrakisphosphate
M00131	Carbohydrate and lipid metabolism	Lipid metabolism	Inositol phosphate metabolism, Ins(1,3,4,5)P4 => Ins(1,3,4)P3 => myo-ii	hsa00562	hsa00562	http://www.genome	C01272: 1D-myo-Inositol 1,3,4,5-tetrakisphosphate	C00137: myo-Inositol
M00132	Carbohydrate and lipid metabolism	Lipid metabolism	Inositol phosphate metabolism, Ins(1,3,4)P3 => phytate	hsa00562	hsa00562	http://www.genome	C01243: 1D-myo-Inositol 1,3,4-trisphosphate	C01204: Phytic acid
M00133_1	Nucleotide and amino acid metabolism	Polyamine biosynthesis	Polyamine biosynthesis, arginine => agmatine => putrescine => spermid	hsa00330	hsa00330	http://www.genome	C00062+C00019: L-Arginine + S-Adenosyl-L-methionine	C00315: Spermidine
M00134	Nucleotide and amino acid metabolism	Polyamine biosynthesis	Polyamine biosynthesis, arginine => ornithine => putrescine	hsa00330	hsa00330	http://www.genome	C00062: L-Arginine	C00134: Putrescine

M00135	Nucleotide and amino acid metabolism	Polyamine biosynthesis	GABA biosynthesis, eukaryotes, putrescine => GABA	hsa00330	hsa00330	http://www.genome	C00134: Putrescine	C00334: 4-Aminobutanoate
M00141	Nucleotide and amino acid metabolism	Cofactor and vitamin biosynthesis	C1-unit interconversion, eukaryotes	hsa00670	hsa00670	http://www.genome	C00101: Tetrahydrofolate	C00101: Tetrahydrofolate
M00338_1	Nucleotide and amino acid metabolism	Cysteine and methionine metabolism	Cysteine biosynthesis, homocysteine + serine => cysteine	hsa00270	hsa01230;hsa00270	http://www.genome	C00065+C00155: L-Serine + L-Homocysteine	C00097: L-Cysteine
M00367	Nucleotide and amino acid metabolism	Terpenoid backbone biosynthesis	C10-C20 isoprenoid biosynthesis, non-plant eukaryotes	hsa00900	hsa00900	http://www.genome	C00129: Isopentenyl diphosphate	C00353: Geranylgeranyl diphosphate
M00415	Nucleotide and amino acid metabolism	Fatty acid metabolism	Fatty acid biosynthesis, elongation, endoplasmic reticulum	hsa00062	hsa01212;hsa00062	http://www.genome	C00083: Malonyl-CoA	C20876: Very-long-chain acyl-CoA
M00549	Carbohydrate and lipid metabolism	Other carbohydrate metabolism	Nucleotide sugar biosynthesis, glucose => UDP-glucose	hsa00520	hsa00520	http://www.genome	C00267: alpha-D-Glucose	C00029: UDP-glucose
M00554	Carbohydrate and lipid metabolism	Other carbohydrate metabolism	Nucleotide sugar biosynthesis, galactose => UDP-galactose	hsa00520	hsa00520;hsa00052	http://www.genome	C00124: D-Galactose	C00052: UDP-alpha-D-galactose
M00632_1	Carbohydrate and lipid metabolism	Other carbohydrate metabolism	Galactose degradation, Leloir pathway, galactose => alpha-D-glucose-1F	hsa00052	hsa00052	http://www.genome	C00124: D-Galactose	C00103: D-Glucose 1-phosphate
M00741	Carbohydrate and lipid metabolism	Other carbohydrate metabolism	Propanoyl-CoA metabolism, propanoyl-CoA => succinyl-CoA	hsa00280	hsa01200;hsa00280;hsa00630;hsa00640	http://www.genome	C00100: Propanoyl-CoA	C00091: Succinyl-CoA

*http://www.genome.jp/kegg-bin/get_htext

eramide

	BRCA			LIHC			KIRC			PRAD		
	Metabolizer	PIA	UGSEA	Metabolizer	PIA	UGSEA	Metabolizer	PIA	UGSEA	Metabolizer	PIA	UGSEA
**Pyruvate (M00001_C00022)**	Up (3.45e-06)						Up (5.6e-07)					
**Pyruvate (M00002_C00022)**	Up (6.55e-05)									Down (0.00738)		
**beta-D-Fructose 6-phosphate (M00003_C05345)**	Up (1.02e-07)			Down (9.51e-11)			Down (1.45e-17)					
**Pentose phosphate cycle (M00004)**				Up (1.7e-10)			Up (4.9e-05)			Up (5.97e-07)		
**D-Ribulose 5-phosphate (M00006_C00199)**	Down (6.4e-28)			Up (0.0361)			Up (8e-07)					
**D-Ribose 5-phosphate (M00007_C00117)**				Up (1.66e-09)						Up (9.6e-09)		
**Citrate cycle (TCA cycle, Krebs cycle) (M00009)**							Down (1.55e-29)		Unknown (0.0108)			
**2-Oxoglutarate (M00010_C00026)**				Up (0.0144)			Down (6.56e-26)		Unknown (0.000315)	Up (0.0234)		
**Oxaloacetate (M00011_C00036)**	Up (7.72e-05)			Down (0.005)			Down (8.85e-31)		Unknown (0.0026)			
**Acetyl-CoA (M00013_C00024)**	Down (2.48e-37)		Unknown (0.0228)	Down (3.83e-22)		Unknown (0.00552)	Down (5.31e-32)		Unknown (0.000125)			
**D-Xylulose 5-phosphate (M00014_C00231)**	Up (1.72e-24)			Down (5.24e-15)			Down (5e-11)			Up (1.32e-15)		
**L-Proline (M00015_C00148)**	Up (1.61e-61)			Up (4.64e-13)			Up (3.09e-07)			Up (2.73e-12)		
**L-Serine (M00020_C00065)**	Down (3.78e-08)			Down (1.5e-06)			Down (1.67e-28)			Up (0.00707)		
**Succinate (M00027_C00042)**	Up (5.35e-10)			Up (7.34e-11)			Down (4.11e-30)			Up (2.84e-12)		
**Fumarate (M00029_C00122)**	Down (0.000997)			Down (1.12e-08)			Down (1.16e-09)			Down (0.0179)		
**Urea (M00029_C00086)**	Down (1.35e-08)			Down (1.85e-12)			Down (8.28e-30)					
**Acetoacetyl-CoA (M00032_C00332)**	Down (1.78e-58)			Down (8.94e-20)			Down (6.59e-27)		Unknown (0.00111)	Up (3.38e-09)		
**Adenine (M00034_C00147)**	Down (2.55e-27)			Down (0.000766)			Down (0.000527)					
**L-Cystathionine (M00035_C02291)**	Up (3.81e-15)			Down (4.32e-12)			Down (1.1e-07)			Up (3.88e-12)		
**Acetoacetate (M00036_C00164)**			Unknown (0.0228)	Down (4.68e-14)		Unknown (9e-05)	Down (1.81e-32)		Unknown (3.78e-05)	Up (3.63e-05)		
**Melatonin (M00037_C01598)**	Down (0.0201)						Up (2.2e-11)	Up (0.00835)		Up (0.0447)		
**L-Adrenaline (M00042_C00788)**				Down (3.6e-15)			Down (1.7e-22)			Down (5.72e-07)		
**Triiodothyronine (M00043_C02465)**	Down (2.06e-54)			Down (3.3e-26)			Down (0.00211)			Up (9.92e-08)		
**Acetoacetate (M00044_C00164)**	Down (1.38e-13)			Down (7.34e-11)			Down (2.61e-11)			Down (3.87e-06)		
**Fumarate (M00044_C00122)**	Down (1.38e-13)			Down (7.34e-11)			Down (2.61e-11)			Down (3.87e-06)		
**beta-Alanine (M00046_C00099)**	Down (5.23e-15)			Down (8.94e-20)					Unknown (0.00502)	Down (0.00126)		
**(R)-3-Amino-2-methylpropanoate (M00046_C01205)**	Down (5.23e-15)			Down (8.94e-20)					Unknown (0.00502)	Down (0.00126)		
**Creatinine (M00047_C00791)**							Down (2.28e-13)			Down (2.2e-16)		
**IMP (M00048_C00130)**	Up (7.91e-39)			Up (8.55e-10)						Up (5.21e-16)		
**ATP (M00049_C00002)**	Up (4.6e-15)			Up (0.000133)						Up (5.96e-07)		
**GTP (M00050_C00044)**	Up (3.63e-39)			Up (8.85e-21)			Up (7.13e-25)			Up (3.1e-13)		
**UMP (M00051_C00105)**	Up (4.33e-27)		Unknown (0.035)				Up (0.0156)		Unknown (0.0013)	Up (1.96e-10)		Unknown (0.0233)
**CDP (M00052_C00112)**	Up (1.13e-47)		Unknown (0.0228)	Up (2.75e-06)			Up (4.93e-15)		Unknown (0.00114)	Up (2.35e-14)		Unknown (0.0216)
**(Glc)3 (GlcNAc)2 (Man)9 (PP-Dol)1 (M00055_G00008)**	Up (7.97e-16)									Up (2.98e-09)		
**Modified serine residues of proteins (M00056_G00025)**	Up (0.000318)			Up (0.00147)						Up (7.16e-13)		
**Modified serine residues of proteins (M00056_G00029)**	Up (5.59e-17)			Up (1.11e-16)			Up (0.00142)			Up (0.000354)		
**Modified serine residues of proteins (M00056_G00031)**	Up (0.00115)			Down (2.76e-05)			Up (2.97e-09)			Up (1.32e-15)		
**Modified serine residues of proteins (M00057_G00157)**	Up (7.51e-28)			Up (1.21e-10)			Up (9.71e-19)		Unknown (0.000263)	Up (3.41e-11)		
**Modified serine residues of proteins (M00058_G00160)**	Up (1.85e-17)			Down (0.00303)			Up (7.82e-27)		Unknown (0.000263)	Up (3.48e-09)		
**Modified serine residues of proteins (M00059_G00164)**	Down (6.59e-18)			Up (1.65e-08)			Down (0.0239)			Down (5.06e-05)		
**Glycosylphosphatidylinositol (M00065_G13044)**	Up (9.3e-20)									Up (1.1e-15)		
**Lactosylceramide (M00066_C01290)**	Down (1.68e-13)						Down (1.26e-11)			Down (0.000234)		
**Sulfatide (M00067_C06125)**	Down (1.41e-28)			Up (0.00101)			Up (0.0236)			Down (2.84e-12)		
**Seminolipid (M00067_C20825)**												
**Globoside (M00068_G00094)**	Up (0.0022)			Up (7.84e-10)			Up (5.41e-05)			Down (1.12e-14)		
**Ganglioside (M00069_G00118)**	Down (5.83e-30)			Up (0.0042)			Up (1.31e-05)			Down (1.5e-12)		
**Lc4Cer (M00070_G00037)**	Down (2.34e-18)			Down (0.0195)			Down (6.64e-05)			Down (1.1e-15)		
**Paragloboside (M00071_G00050)**	Down (2.98e-17)			Up (0.0015)			Up (1.3e-16)			Down (0.000461)		
**Modified asparagine residues of proteins (M00073_G00012)**	Up (1.98e-23)						Down (4.9e-05)			Up (7.66e-14)		
**Modified asparagine residues of proteins (M00075_G00019)**	Down (1.46e-35)			Up (8.94e-09)			Up (3.71e-14)			Down (0.00485)		
**Modified asparagine residues of proteins (M00075_G00022)**	Down (6.95e-19)	Down (0.00459)					Down (1.68e-31)	Down (0.00835)		Down (2.92e-14)		
**Modified asparagine residues of proteins (M00075_G00018)**	Up (7.72e-05)			Down (3.15e-05)			Down (1.2e-13)					
**Chondroitin 4-sulfate (M00076_G00872)**	Up (4.95e-08)						Up (1.14e-26)			Up (4.36e-05)		
**Chondroitin 4-sulfate (M00077_G00872)**	Up (2.46e-05)											
**Product of heparan sulfate degradation (M00078_G02632)**							Up (1.42e-09)					
**Product of keratan sulfate degradation (M00079_G01391)**	Up (4.64e-24)						Down (0.00758)					
**Acetoacetyl-[acp] (M00082_C05744)**	Down (1.81e-07)			Up (0.000125)						Up (6.29e-20)		
**Butyryl-[acp] (M00083_C05745)**	Up (2.19e-09)			Up (1.66e-09)						Up (2.93e-22)		
**Acyl-CoA (M00085_C00040)**	Down (4.74e-42)			Down (2.62e-14)			Down (2.22e-20)					
**Tetradecanoyl-CoA (M00087_C02593)**	Down (1e-51)		Unknown (0.00663)	Down (3.3e-19)		Unknown (1.51e-06)	Down (9.95e-20)		Unknown (0.000681)			
**Triacylglycerol (M00089_C00422)**	Down (6.65e-38)			Down (4.64e-13)			Down (1e-19)			Up (9.44e-08)		
**Phosphatidylcholine (M00090_C00157)**	Down (4.19e-15)			Up (0.0019)			Down (3.05e-12)			Down (0.00485)		
**Phosphatidylcholine (M00091_C00157)**	Down (1.44e-13)			Down (6.37e-19)						Up (0.000677)		
**Phosphatidylethanolamine (M00092_C00350)**	Up (4.7e-26)			Down (0.000525)			Down (4.75e-31)			Up (6.81e-05)		
**N-Acylsphingosine (M00094_C00195)**	Down (1.4e-20)			Down (2.09e-16)			Down (3.57e-06)			Down (3.03e-14)		
**Dimethylallyl diphosphate (M00095_C00235)**	Up (1.53e-19)			Up (0.0342)			Down (1.76e-31)					
**Glycerol (M00098_C00116)**	Down (0.000984)			Down (5.39e-21)			Down (9.93e-08)			Down (5.89e-11)		
**Sphingosine (M00099_C00319)**	Down (2.16e-20)			Down (5.24e-15)			Down (2.41e-10)			Down (8.94e-11)		
**Ethanolamine phosphate (M00100_C00346)**	Up (1.07e-42)						Down (7.29e-25)			Up (3.99e-10)		
**Cholesterol (M00101_C00187)**	Up (1.62e-66)	Up (0.00459)	Unknown (0.0228)	Up (1.1e-26)			Up (3.57e-41)			Up (1.27e-29)		
**Calcitriol (M00103_C01673)**	Up (2.55e-27)		Unknown (0.00663)	Up (7.84e-16)		Unknown (0.00552)	Down (2.78e-13)			Up (1.96e-10)		
**Cholic acid (M00104_C00695)**	Down (2.42e-24)			Down (1.8e-10)						Up (0.0347)		
**Chenodeoxycholate (M00104_C02528)**	Down (8.36e-16)			Down (3.33e-09)								
**Taurocholate (M00106_C05122)**	Up (1.31e-09)			Down (7.22e-06)			Up (4.75e-12)			Up (0.00485)		
**Glycocholate (M00106_C01921)**	Up (1.31e-09)			Down (7.22e-06)			Up (4.75e-12)			Up (0.00485)		
**Progesterone (M00107_C00410)**	Down (3.67e-46)			Down (0.000118)			Down (2.31e-11)			Down (3.03e-08)		

**Aldosterone (M00108_C01780)**	Down (3.17e-12)			Down (1.16e-14)			Up (4.04e-07)					
**Cortisone (M00109_C00762)**				Down (0.00497)			Down (1.16e-12)					
**Estrone (M00110_C00468)**	Down (0.00994)			Up (3.63e-06)	Up (0.0152)					Down (0.00749)	Down (0.0128)	
**Glutathione (M00118_C00051)**				Down (0.00139)						Down (0.0186)		
**CoA (M00120_C00010)**	Up (9.74e-31)						Down (1.74e-16)			Up (6.62e-07)		
**Ubiquinone (M00128_C00399)**	Up (1.31e-09)			Down (1.33e-10)			Down (3.99e-25)			Up (1.77e-07)		
**1D-myo-Inositol 1,3,4,5-tetrakisphosphate (M00130_C01272)**				Up (1.39e-14)			Down (3.5e-10)			Down (4.35e-05)		Unknown (0.0233)
**myo-Inositol (M00131_C00137)**	Down (5.43e-42)			Down (0.0223)			Up (2.21e-12)			Down (5.97e-12)		Unknown (0.0233)
**Phytic acid (M00132_C01204)**	Up (0.000437)			Down (0.000525)								Unknown (0.0233)
**Spermidine (M00133_C00315)**				Up (0.0315)			Up (7.28e-19)			Up (2.94e-13)		
**Putrescine (M00134_C00134)**	Down (9.62e-16)			Down (0.000205)			Down (8.28e-29)					
**4-Aminobutanoate (M00135_C00334)**	Down (3.91e-42)			Down (3.35e-16)			Down (2.27e-07)			Down (0.0138)		
**C1-unit interconversion (M00141)**	Up (0.0167)			Down (2.63e-17)			Down (0.00222)			Up (3.03e-14)		
**L-Cysteine (M00338_C00097)**	Up (2.11e-09)			Down (7.38e-16)			Down (3.48e-29)			Up (9.44e-11)		
**Geranylgeranyl diphosphate (M00367_C00353)**	Up (1.22e-40)			Up (2.88e-11)			Down (1.18e-10)			Up (0.000693)		
**Very-long-chain acyl-CoA (M00415_C20876)**	Up (1.27e-61)			Up (1.49e-26)			Up (1.06e-40)			Up (1.62e-28)		
**UDP-glucose (M00549_C00029)**	Down (7.92e-31)			Down (1.23e-05)			Up (6.14e-14)			Down (9.92e-08)		
**UDP-alpha-D-galactose (M00554_C00052)**							Down (0.00149)					
**D-Glucose 1-phosphate (M00632_C00103)**	Up (0.0346)			Down (0.000985)			Down (6.04e-10)					
**Succinyl-CoA (M00741_C00091)**			Unknown (0.0228)	Down (2.14e-20)		Unknown (3.66e-05)	Down (1.21e-33)		Unknown (1.26e-05)	Up (0.000249)		

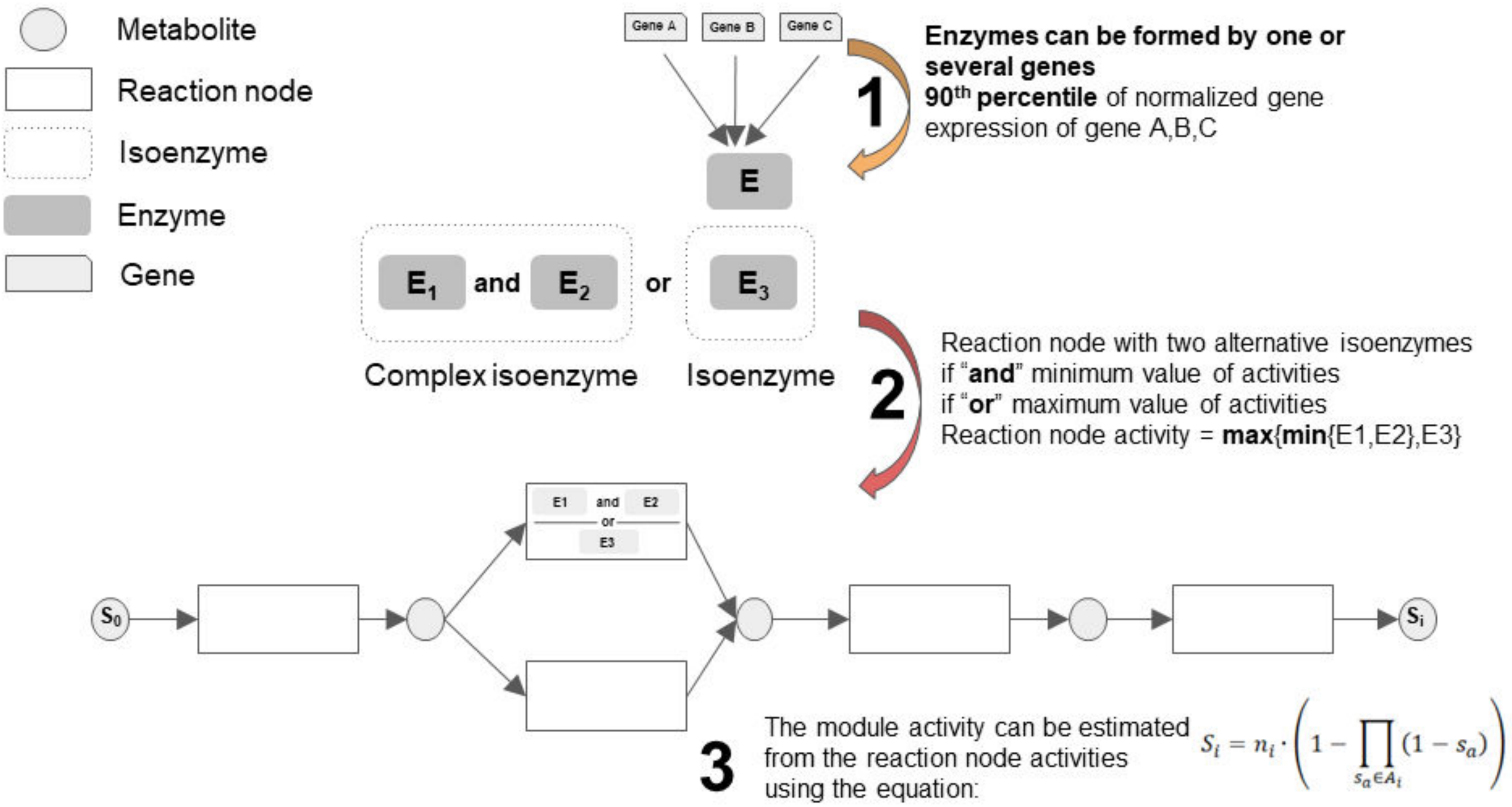
Metabolite
Reaction node
Isoenzyme
Enzyme
Gene
Gene A
Gene B
Gene C
E
1
Enzymes can be formed by one or several genes
90th percentile of normalized gene expression of gene A,B,C
E1 and E2 or E3
Complex isoenzyme
Isoenzyme
2
Reaction node with two alternative isoenzymes
if "and" minimum value of activities
if "or" maximum value of activities
Reaction node activity = max{min{E1,E2},E3}
E1 and E2
or
E3
S0
Si
3
The module activity can be estimated from the reaction node activities using the equation:
$S_i = n_i \cdot \left(1 - \prod_{s_a \in A_i} (1 - s_a)\right)$

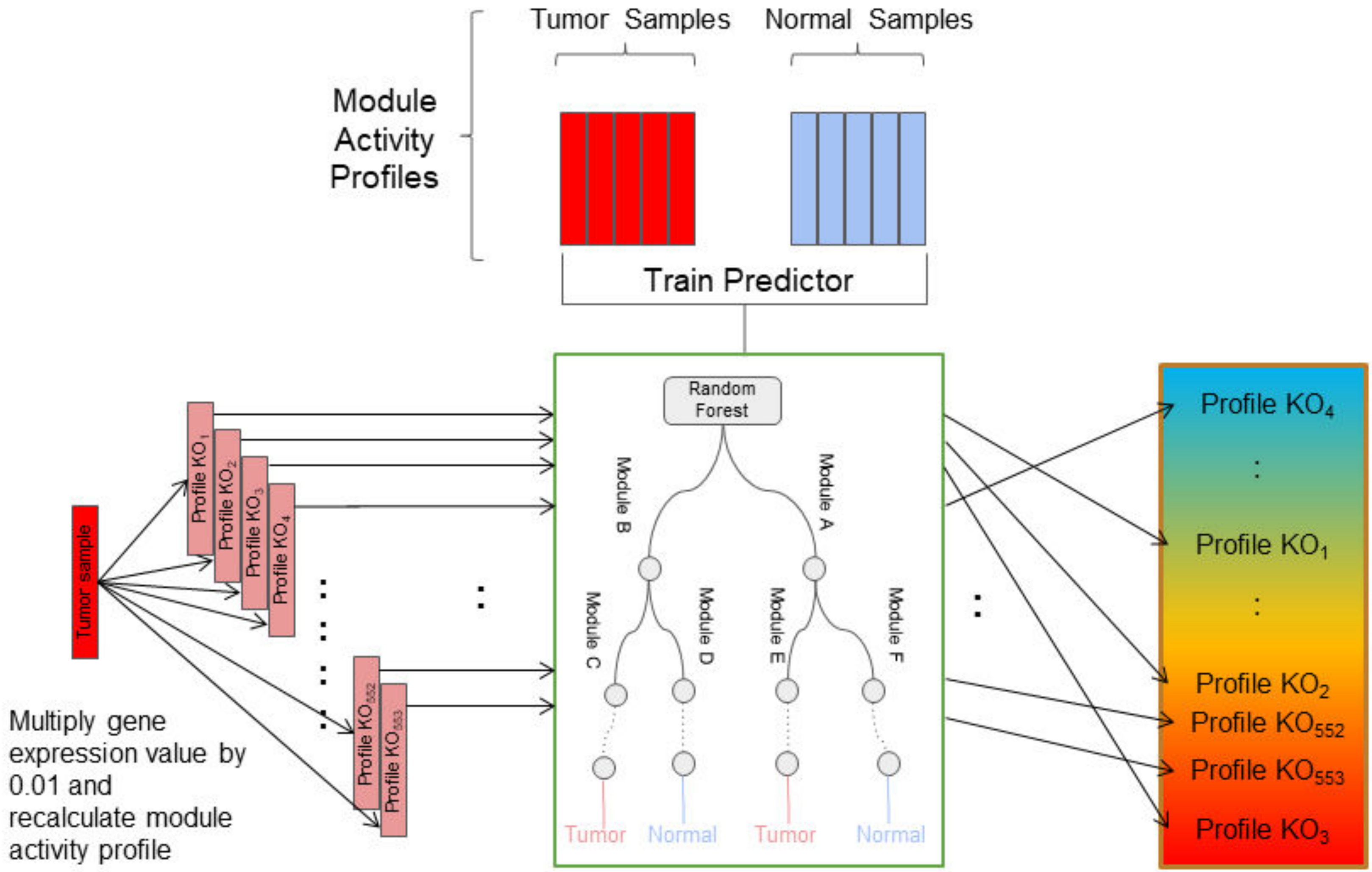

Tumor Samples
Normal Samples
Module Activity Profiles
Train Predictor
Random Forest
Module B
Module A
Module C
Module D
Module E
Module F
Tumor
Normal
Tumor
Normal
Tumor sample
Profile KO1
Profile KO2
Profile KO3
Profile KO4
Profile KO552
Profile KO553
Multiply gene expression value by 0.01 and recalculate module activity profile
Profile KO4
Profile KO1
Profile KO2
Profile KO552
Profile KO553
Profile KO3
